# Recent Advances in Exosome-Based Drug Delivery for Cancer Therapy

**DOI:** 10.3390/cancers13174435

**Published:** 2021-09-02

**Authors:** Hyosuk Kim, Hochung Jang, Haeun Cho, Jiwon Choi, Kwang Yeon Hwang, Yeonho Choi, Sun Hwa Kim, Yoosoo Yang

**Affiliations:** 1 Center for Theragnosis, Biomedical Research Division, Korea Institute of Science and Technology (KIST), Seoul 02792, Korea; hyoseog7@kist.re.kr (H.K.); hjang@kist.re.kr (H.J.); 120047@kist.re.kr (H.C.); c9307c@kist.re.kr (J.C.); 2 Division of Bio-Medical Science and Technology, KIST School, Korea University of Science and Technology, Seoul 02792, Korea; 3 Department of Biotechnology, College of Life Sciences & Biotechnology, Korea University, Seoul 02841, Korea; chahong@korea.ac.kr; 4 Department of Bioengineering, Korea University, 145 Anam-ro, Seongbuk-gu, Seoul 02841, Korea; yeonhochoi@korea.ac.kr

**Keywords:** exosome, drug delivery system, cancer therapy, exosome engineering

## Abstract

**Simple Summary:**

Exosomes derived from various sources can deliver therapeutic agents such as small molecule drugs, nucleic acids, and proteins to cancer cells by passive or active targeting. These exosomes can encapsulate drugs inside the exosomes, extending drug half-life and increasing drug release stability. In addition, exosomes are highly biocompatible due to their endogenous origin and can be used as nanocarriers for tissue-specific targeted delivery. This review discusses recent advances in exosome-based drug delivery for cancer therapy.

**Abstract:**

Exosomes are a class of extracellular vesicles, with a size of about 100 nm, secreted by most cells and carrying various bioactive molecules such as nucleic acids, proteins, and lipids, and reflect the biological status of parent cells. Exosomes have natural advantages such as high biocompatibility and low immunogenicity for efficient delivery of therapeutic agents such as chemotherapeutic drugs, nucleic acids, and proteins. In this review, we introduce the latest explorations of exosome-based drug delivery systems for cancer therapy, with particular focus on the targeted delivery of various types of cargoes.

## 1. Introduction

Exosomes, which are membrane structures surrounded by a lipid bilayer with a diameter of about 100 nm, have recently attracted much attention as novel drug delivery nanoplatforms. Exosomes are known as transporters that carry cargos such as nucleic acids, proteins, and lipids as part of the interaction between the parent cell and the distant or adjacent cells to respond to the external environment [1]. Exosomes are one of the extracellular vesicles that originated from the endosomal compartment. However, recent studies have reported that the small-size microvesicles (around 100 nm) can also be included in the “exosome” category [2,3]. As there is no molecular marker to differentiate multivesicular body-derived exosomes from other extracellular vesicles, in this review, the term “exosome” is used to refer to small-size extracellular vesicles.

The composition of exosomes can represent the biological state of the cells from which they are derived and has been implicated in the physiological and pathological processes of various diseases. In particular, it is known to play an important role in the pathogenesis of cancer, including the communication between multiple cells such as cancer cells and immune cells [4,5].

**Figure 1:**
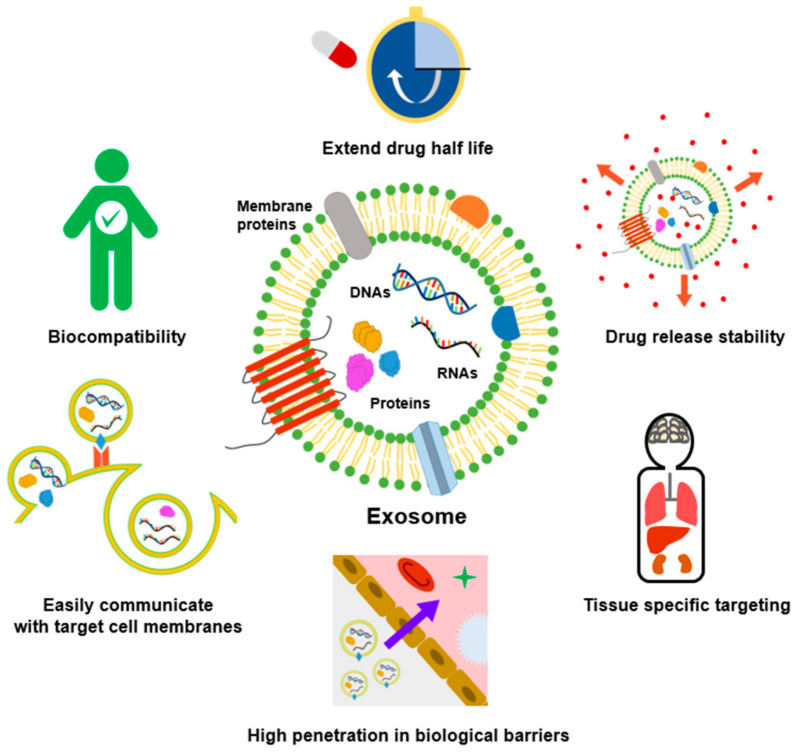
Advantages of exosomes in cancer treatment compared to other nanoparticles. Exosomes with lipid bilayer membrane structures encapsulate the drug inside, thereby extending drug half-life and increasing drug release stability. In addition, exosomes can easily communicate with target cell membranes and cross biological barriers such as the BBB. Due to their endogenous origin, they are highly biocompatible and can be used as nanocarriers for tissue-specific targeted delivery.

Despite their involvement in tumor progression and metastasis, exosomes obtained from both healthy and diseased cells could be utilized as drug delivery vehicles, immunomodulators, etc. [6,7,8]. Exosomes have many advantages compared to synthetic nanoparticles (Figure 1). Compared with conventional liposomes and artificial nanoparticles, exosomes have high biocompatibility and cellular uptake due to membrane proteins such as tetraspanin and fibronectin, and can be easily modified according to target cells [9,10]. Exosomes are more stable in body fluids than liposomes with similar structures and characteristics. For example, liposomes can be easily removed directly or indirectly by macrophages or reticuloendothelial cells [11] but exosomes are known to be highly biocompatible because of their endogenous origin. In addition, abundant studies have suggested that exosomes could evade the immune system and prolong circulation time in the body [12,13,14]. Moreover, due to their small size, it can overcome biological barriers, such as the blood–brain barrier (BBB) and lung clearance, and drugs with low stability such as curcumin can be loaded into the exosomes to increase their stability [15,16,17].

Exosomes show very high cell uptake efficiency via directly interacting with the extracellular proteins or the direct membrane fusion or internalization [18]. Exosomes derived from a variety of cellular sources also carry diverse membrane proteins [19]. Integrin, which are lipid raft-like domains on the surface of exosomes derived from each specific source, mediate membrane fusion and endocytosis through interactions with target cells [9,20]. Through these target cell-specific surface proteins, exosomes have superior targeting ability compared to artificial nanoparticles [21].

Besides membrane proteins, exosomes inherit bioactive molecules that may have therapeutic benefits from donor cells. For example, M2 macrophage-derived exosomes accelerated wound healing by delivering anti-inflammatory cytokines derived from their parental cells to a mouse wound healing model [22]. Milk-derived exosomes that contained immune-related miRNAs showed excellent therapeutic efficacy in an inflammatory bowel disease mouse model by oral delivery [23]. MSC-derived exosomes could modulate immunity and promote tissue regeneration but could also accelerate the growth of tumors by stimulating factors related to angiogenesis [24]. Therefore, careful donor cell selection is essential for drug delivery by exosomes for cancer therapy.

**Figure 2:**
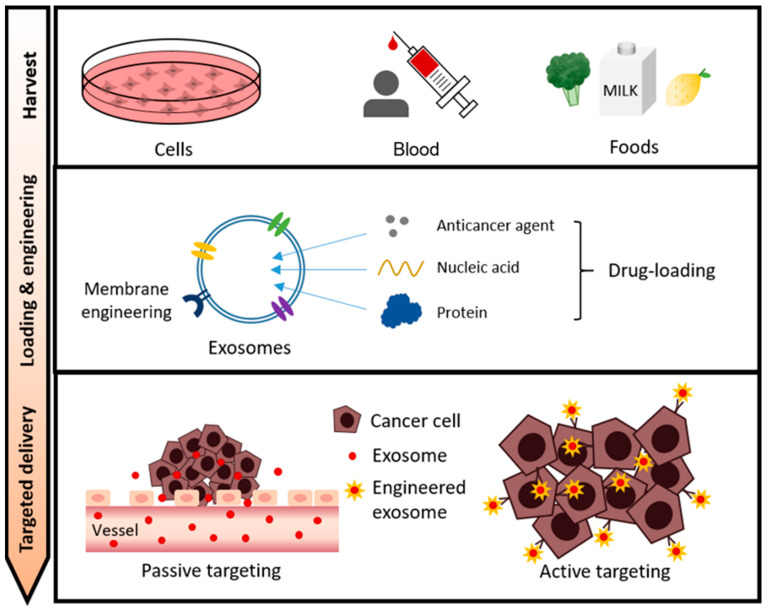
The overall process of exosome-based drug delivery in cancer therapy. Cell/blood/food-derived exosomes can be sources for drug delivery carriers. Exosomes loaded with therapeutic agents such as anticancer agents, nucleic acids, and proteins can be delivered to cancer cells by passive or active targeting.

Sources of exosomes widely used as drug carriers include cells, blood (body fluids), and food. Therapeutic cargos for cancer therapy such as anticancer drugs, nucleic acids, and proteins are loaded into exosomes harvested from these sources through various loading methods. In addition, for efficient drug delivery, exosomes are being optimized through various exosome surface engineering. In this review, we discuss a state-of-the-art exosome-based drug delivery system for cancer treatment depending on the exosome source, types of cargoes, and exosomal surface engineering (Figure 2).

## 2. Main Types of Exosomes in Drug Delivery Systems

As the field of research on effective drug delivery systems (DDS) development has been grown eagerly, the exosomes possessing low immunogenicity and a high bio-absorption rate are being focused as potent drug carriers. The main purpose of the DDS is to allow the drug to overcome biological barriers (e.g., cell membrane, efflux transporter, and metabolic enzymes) to have high bioavailability in target regions [25,26,27,28].

The physicochemical properties affecting pharmacokinetics of exosomes may differ depending on the type of source of exosomes [29,30]. Therefore, it is important to study the ways in which different biochemical features of exosomes are derived from various sources. In this section, we will focus on categorizing the different types of exosomes derived from different origins (Table 1).

### 2.1. Cell-Secreted Exosomes

Basically, exosomes, which are a type of nano-sized extracellular vesicle (EV), are released by almost any type of cell [2,52,53]. Considering EVs are heterogeneous in size, content, and function, it is difficult to distinguish exosomes from other EVs such as microvesicles. In the current EV field, compared to exosomes isolated from complex biological fluids such as plasma, methods for characterizing exosomes isolated from conditioned cell culture media are relatively well established. The known “gold standard” isolation of exosomes as a subset of EVs is differential centrifugation, which typically consists of low-speed centrifugation to remove cells and large vesicles, and high-speed ultracentrifugation to extract only exosomes [54]. In addition, ultrafiltration, density gradient purification, polymer precipitation, size exclusion purification, and immunoaffinity chromatography are widely used for various purposes in exosome applications [55]. Many research groups have been extracting exosomes from human embryonic kidney (HEK) cells, cancer cells, immune cells, and stem cells, and these exosomes have individual characteristics according to their origin.

#### 2.1.1. Human Embryonic Kidney Cell (HEK)-Derived Exosomes

The HEK cell line (HEK293T) is the most commonly applicated cell line in the field of biopharmaceutical manufacturing due to its advantageous properties such as ease of growth, non-demanding maintenance conditions, and high transfection efficiency [31].

According to some previous studies, exosomes isolated from HEK293T have membrane resemblances to various tissues in our body (e.g., epithelium, skin, brain, liver, lung, muscle, lymph, and hepatocytes) [32,56,57,58]. This suggests that HEK-derived exosomes enable drug delivery to various target tissues. In addition, Zhu et al. reported that repeated dosing of exosomes obtained from HEK293T for 3 weeks did not induce any noteworthy adverse effects in terms of the immune response and toxicity in mice [31]. In addition to targeting and safety-related properties, HEK-derived exosomes can also increase drug delivery and therapeutic efficiencies by delivering fully functional natural forms of membrane proteins to cancer cells. Kim et al. employed genetically modified HEK exosomes for tumor cell “xenogenization”. The authors transfected plasmids encoding mutant vesicular stomatitis virus glycoprotein (mVSVG) into HEK293T cells to harvest mVSVG-engineered exosomes (mVSVG-Exo). mVSVG-Exo increased bone marrow-derived macrophages (BMDMs) and dendritic cell (BMDC)-mediated phagocytosis of xenogenized cancer cells [59]. Moreover, it was found that therapeutic membrane protein-expressing exosomes can also improve tumor penetration and antitumor efficacy. As seen in experimental results by Hong et al., native PH20 hyaluronidase-expressing exosomes obtained from HEK293T cells inhibit tumor growth by degrading hyaluronan in the tumor extracellular matrix (ECM), the primary component of the tumor microenvironment. In addition, co-delivery of PH20 and doxorubicin (Dox) showed markedly enhanced antitumor effects compared to Dox-only delivery groups in the tumor-bearing mouse model [60].

#### 2.1.2. Cancer Cell-Derived Exosomes

Since cancer cell lines overexpressed two subtypes of Rab proteins (Rab27a and Rab27b) involved in the process of exosome release [61], cancer cells are also considered as efficient exosome producers. One of the most representative characteristics of cancer cell-derived exosomes is a tropism toward their parent cells [33,35]. Indeed, Qiao and colleagues isolated exosomes from two different cancer cell lines (HT1080, a fibrosarcoma cell line, and HeLa, a cervical cancer cell line) and confirmed that exosomes derived from HT1080 showed twice as much uptake in HT1080 cells compared to HeLa exosomes. Furthermore, the authors conducted an in vivo efficacy test using anticancer drug-encapsulating HT1080 exosomes and found that drug-loading HT1080 exosomes showed significantly increased accumulation at HT1080 tumor site versus HeLa exosomes [35]. Although cancer cell-derived exosomes have shown promising ability as drug delivery vehicles, there are some limitations to be improved to utilize them for cancer treatment. First, cancer cell-produced naive exosomes have a less ideal pharmacokinetic profile [34]. Second, based on many studies regarding that cancer exosomes may be involved in tumor metastasis, it is necessary to fully consider the possible adverse effects when using cancer exosomes as drug carriers [35]. If the aforementioned shortcomings are improved, it is expected that exosomes isolated from cancer patients can be used as a good weapon to treat them.

#### 2.1.3. Immune Cell-Derived Exosomes

Dendritic cells (DCs) that present tumor antigens to naive T cells are widely used for T cell-mediated immunotherapy but have the disadvantage of a short lifespan after activation [62]. However, the DC-derived exosomes (DEX) are proposed as a key molecule to complement the limitations of DC-based immunotherapy because DEX preserve the immune stimulation-related abilities of their origin [40]. First, DEX has a strictly defined molecular composition corresponding to each patient, which determines the molecular parameters for quality control in biologics [37,40]. Second, the lipid composition of DEX membranes facilitate long-term storage at −80 °C [40]. Considering that DC-based therapy employs living cells, DEX show improved stability compared to DCs in the preparation process of therapeutics. Third, DEX express the ligand peptides that activate NK cells on their surface [36,38,39]. In addition, DEX has 10-to-100-fold more abundant MHCII molecules compared to DC, allowing for DEX therapy up to 6 months with a single leukapheresis [37,40]. In general, DEX isolated from immature DCs (imDCs) showed a lack of immunostimulatory molecules’ expression (e.g., CD86 and CD40), which can prevent unexpected immune responses induced by naive T cell stimulation [63].

#### 2.1.4. Stem Cell-Derived Exosomes

Among various cell types known to secret exosomes, mesenchymal stem cells (MSCs) are also considered an ideal source to prepare exosomes for clinical application in that they can be isolated from a variety of human tissues and have a large capacity for ex vivo expansion [64,65]. The kalluri group developed a scaled-up isolation process for the production of good manufacturing practice (GMP)-grade exosomes from bone marrow-derived MSCs for clinical use. Using this isolation method and MSCs, the authors obtained a three-fold increase of quantities of exosomes compared to those from human foreskin fibroblasts (BJ fibroblasts), which have a similar morphology and surface marker with MSCs. In addition, the GMP-grade MSC exosomes can deliver therapeutic siRNA targeting oncogenic Kras (MSCs siKras Exo). After administration of MSCs siKras Exo, tumor size and metastatic level were significantly decreased in the tumor-bearing mouse model [66].

### 2.2. Blood (Red Blood Cell)-Derived Exosomes

Basically, exosomes contain many types of biological molecules characterized by their parent cells, such as nucleic acids, proteins, and lipids [67,68]. Thus, exosomes isolated from body fluids including blood plasma, urine, saliva, and amniotic fluids have been applied to the diagnosis of several diseases [69,70,71,72,73]. Among these types of exosomes from different body fluids, blood (red blood cell)-derived exosomes have been used for the delivery of nucleic acid-based therapeutics [43].

According to the previous study from the Le group, red blood cell (RBC)-derived exosomes were suggested as a versatile delivery vehicle for therapeutic RNAs. RBC-derived exosomes have several advantageous properties for clinical application. Briefly, (1) blood units, the source of exosomes, can be readily obtained from blood banks and patients, themselves, as required. (2) Considering a relatively large amount of RBC (~10^12^ cells/L) exists in each blood unit [74], the risk of unexpected in vitro mutations that may occur during cell culture can be reduced. (3) Unlike other cells with a nucleus, RBCs are enucleated cell types [75]. Therefore, this suggests that exosomes isolated from RBCs are free from gene-related potential risks such as horizontal gene transfer. (4) Similar to blood transfusion, RBC-derived exosomes can be prevented from inducing toxic and immunogenic responses by matching blood types between donors and recipients. (5) RBC exosomes provide higher transfection efficiency. To evaluate the transfection efficiency of RBC exosome-based RNA therapeutics delivery, researchers encapsulated 400 pmol of FAM-labeled antisense oligonucleotides (ASOs) into RBC exosomes and incubated it with leukemia MOLM13 cells for 24 h. By quantifying a percentage of FAM-positive cells using FACS analysis, it was verified that RBC exosomes showed an approximately two-fold increase in transfection efficiency (80.4%) compared to commercial transfection reagents such as Lipofectamine^TM^3000 (41.0%) and INTERFERin^®^ (31.3%) which have a lower toxicity [43].

### 2.3. Food-Derived Exosomes

In addition to the typical limitations of cell-derived exosomes such as small amounts of yield and the possibility of inducing immunogenicity, the limited dosing route is also suggested as an improvement point. To this end, food, such as milk and edible plants, are proposed as an alternative source of exosomes due to its various beneficial features for clinical application [76].

#### 2.3.1. Milk-Derived Exosomes

Since milk, a type of body fluid, contains various supporting ingredients for growth, it is consumed by not only newborns but also adults [77,78]. In particular, bovine-derived milk is emerging as a promising substitute for an exosome source in that it can be mass-produced (according to a report from the US Department of Agriculture (USDA), the average annual milk production per cow in 2020 is approximately 24,000 pounds) [79]. The main advantage of milk-derived exosomes is that they enable the effective delivery of encapsulated therapeutic molecules through the oral cavity due to its stability under low pH-degrading gastric conditions [45,46,48]. Exosomes derived from bovine’s milk can elicit cross-species transportation through conserved IgG-neonatal Fc receptor (FcRn), binding in the upper gastrointestinal tract [76,80,81,82,83]. Agrawal et al. reported that orally administered paclitaxel (PTX)-loaded exosomes (ExoPAC) markedly inhibited tumor growth in the tumor-bearing mouse model without any adverse effects on systemic toxicity and immune responses [80]. In addition to biocompatibility and safety perspectives, milk exosomes can also be functionalized through post-isolation modification. A research group, led by Bajpayee et al., has engineered milk exosomes using polyethylene glycol (PEG) to improve their integrity in acidic gastric environments and mucus permeability. As expected, PEG-modified milk exosomes showed an approximately 3.2-fold increase in mucus permeability compared to unmodified milk exosomes [84].

#### 2.3.2. Edible Plant-Derived EXOSOMES

Considering that exosomes isolated from plants can be administered orally and modified with functional moieties such as folate, plant-derived exosomes (PDEs) are also considered as promising candidates of an exosome source. First, it has been reported that PDEs per se have protective effects on inflammatory disease [50,85,86]. For instance, Ju and colleagues found that exosomes isolated from grapes can modulate intestinal homeostasis and have protective effects on dextran sulfate sodium (DSS)-induced colitis after oral administration [50]. According to the previous study from Zhang et al., it was found that folic acid-modified ginger-derived nanovectors (GDNVs), which are exosome-like nanoparticles from edible ginger, have a significantly great biocompatibility compared to cationic liposomes, as well as effective cancer targeting/therapeutics delivery efficiency [49]. Although in vivo studies in this article were conducted via intravenous injection, it is anticipated that the development of less invasive anticancer drug delivery strategies using PDE is also possible considering PDE’s therapeutic effects after oral administration.

## 3. Exosome Cargo Loading for Cancer Therapy

Exosomes are broadly utilized as drug vehicles for various cancer therapeutic cargoes with the advantages described above. As a delivery system, exosomes with lipid bilayer membranes can safely protect and deliver different cargoes, including anticancer small molecule drugs, nucleic acids, and proteins, in a sustained release manner. In this section, the unique advantages and several examples of exosomes for delivering each cancer therapeutic cargo are presented (Table 2).

### 3.1. Anti-Cancer Drugs

Hydrophilic and hydrophobic chemotherapeutic drugs including Dox and PTX have been reported to be loaded in exosomes. A number of accumulating studies have shown that exosome-mediated chemotherapeutic delivery can enhance anti-cancer effects [87,88,89,90,91].

Dox, one of the most effective anti-cancer drugs, is used for the treatment of leukemia, lymphoma, and many types of solid tumors. However, the clinical use of Dox is very limited due to their poor biocompatibility and serious side effects such as bone marrow suppression and cardiotoxicity. Although many efforts are being made to enhance the biocompatibility and anti-cancer effects of Dox through various nanoparticle technology, there have also been nanoparticle-associated side effects that must be overcome, such as immune response and oxidative stress [92,93].

As Dox can be easily tracked by its intrinsic fluorescence, it has been well studied in exosome-mediated anti-cancer therapy. Exosomes generated through serial extrusion from doxorubicin-pretreated macrophages show superior anti-cancer effects than free Dox or Dox-loaded liposome groups in the colon adenocarcinoma mouse model [94]. Compared with liposomes, the ability of exosomes to target cancer cells is quite high due to the optimized mechanism of endocytosis by cholesterol and the phospholipid composition present on the exosomal membranes [95]. Dox-loaded exosomes prevent the delivery of Dox to myocardial endothelial cells, thereby reducing cardiotoxicity, a representative side effect of Dox [96]. More recently, it has been reported that exosomes derived from mesenchymal stem cells could enhance the cellular uptake rate and anticancer effect of Dox in osteosarcoma [97]. This may be due to the tropism of mesenchymal stem cells toward tumor tissues, suggesting the importance of the selection of exosome sources.

PTX is another widely used anti-mitotic agent for malignant tumors such as glioblastoma multiforme and breast cancer [80,98]. PTX is often used to overcome drug resistance in cisplatin-resistant patients. However, PTX has a dose-dependent toxic effect with low bioavailability, which is a major obstacle for clinical application. Additionally, several studies reported that PTX could not pass through BBB [99,100,101]. Mesenchymal stromal cells pretreated with PTX could produce PTX-loaded exosomes, which showed strong anti-cancer effects in human pancreatic adenocarcinoma [102]. Moreover, cancer-derived exosomes encapsulating PTX could directly target drug-resistant cancer stem cells, improving cytotoxicity against autologous cancer cells [103]. Multiple drug resistance (MDR) is one of the major obstacles for successful cancer treatment. Exosomes have been shown to be effective in overcoming MDR in tumors. PTX-loaded macrophage-derived exosomes could bypass the P-glycoprotein drug efflux transporter and showed high cell uptake in MDCK MDR1 cells and lower IC_50_ than free PTX [104]. In addition to overcoming MDR, exosomes derived from U-87 MG cells could pass through the BBB and deliver PTX, thereby having an improved therapeutic effect in glioblastoma multiforme [98].

**Table 2:** Exosome cargo loading for cancer therapy.

Cargo		Origin of Exosomes	Target Cancer Type	Loading Method	Administration Route	Outcome	References
Doxorubicin		Monocyte	Colon carcinoma	Incubation	I.V.	Suppression of tumor growth	[94]
		Breast cancer cell Ovarian cancer cell	Breast cancer Ovarian cancer	Electroporation	I.P.	Suppression of tumor growth Reducing toxicity of drug	[96]
		MSC	Osteosarcoma myocardial	Incubation	N/A	Reducing toxicity of drug Enhanced the cellular uptake rate	[97]
Paclitaxel		MSC	Pancreatic adenocarcinoma	Incubation	N/A	Improved anticancer effect	[102]
		Prostate cancer cell	Prostate cancer	Incubation	N/A	Overcame drug resistance Enhanced cytotoxicity on cancer cells	[103]
		Macrophage	Pulmonary metastasis	Incubation Electroporation Sonication	I.N.	Overcame MDR in cancer cells Enhanced the cellular uptake rate	[104]
		Bovine milk	Lung cancer	Incubation	P.O.	Suppression of tumor growth Lower systemic toxicity	[80]
		Brain cancer cell	Glioblastoma-astrocytoma cancer	Incubation Sonication	N/A	Enhanced cytotoxicity on cancer cells Passed through the BBB	[98,105]
Nucleic acid	miRNA-122	AMSC	Hepatocellular carcinoma	Transfection	I.T.	Improved the chemosensitivity Improved anticancer effect	[106]
	miRNA-21	HEK293T cell	Brain tumor	Electroporation	I.V.	Suppression of tumor growth Passed through the BBB	[107]
	siRNA	Breast cancer cell	Breast adenocarcinoma	Electroporation	I.V.	Suppression of tumor growth Improved effect of tumor targeting	[108]
		Breast cancer cell	Breast cancer Lung metastasis	Incubation	I.V.	Enhanced the cellular uptake rate Reduced migration and metastasis of cancer cells	[109]
	Circular RNA	Hepatocellular cancer cell	Hepatocellular cancer	Pre-overexpression	I.T.	Suppression of tumor growth Promoted tumor cell apoptosis	[110]
	mRNA	Red blood cell	Breast cancer Leukemia	Electroporation	I.T.	Suppression of tumor growth Reduced drug cytotoxicity	[43]
Protein	Surviving-T34A	Melanoma cell	Pancreatic adenocarcinoma	Transfection	N/A	Induced apoptosis in adenocarcinoma cells Enhanced drug sensitivity	[111]
	TRP2	Serum	Any type of cancer	Saponin/electroporation	S.C.	Accumulation in lymph nodes for immunotherapy	[112]
	SIRPα	HEK293T cell	Colon carcinoma	Transfection	I.V.	Enhanced phagocytosis of tumor cells Suppression of tumor growth	[113]
	hMUC1	Colon cancer cell Breast cancer cell	Colon carcinoma	Transfection	I.D.	Proliferation and activation of immune cells Suppression of tumor growth	[114]

Besides PTX, hydrophobic natural compounds such as curcumin are also being studied clinically. Among hydrophobic anti-cancer drugs, curcumin is readily incorporated into the exosomal membranes. According to a recent study, loading curcumin into exosomes derived from intestinal epithelial cells showed potential as an oral drug with improved cellular uptake and intestinal permeability [115].

### 3.2. Nucleic Acids

Gene therapy using nucleic acids such as DNA and RNA is an attractive and promising strategy for cancer treatment. In particular, small RNAs such as siRNA or miRNA generally bind to mRNAs and increase or decrease their activity to regulate gene expression [116,117]. Nano-based delivery systems such as liposomes, cationic polymers, and inorganic nanoparticles have been developed to protect these nucleic acids from degradation by endonuclease and deliver them to tumors [118,119]. However, to apply these gene delivery systems to actual clinical practice, barriers regarding safety, stability, and delivery efficiency need to be overcome. In general, these nanocomplexes are induced by electrostatic interactions between positively charged carriers and the strong negative charge due to the phosphate backbone of RNA. The stability of the complex by charge interaction increases the protection for RNA but makes the release of small RNAs for gene regulation difficult. Moreover, these cationic surface charges of nanocarriers may induce cytotoxicity. Therefore, the balance between the protection and release of small RNAs is a critical factor for efficient delivery of small RNAs [120].

Recently, exosomes are attracting attention as vehicles for gene delivery due to their unique characteristics that can overcome these difficulties. Abundant miRNAs involved in intercellular communication were detected in exosomes [121], some of which exhibit anti-cancer properties. A method of loading desired RNAs into exosomes through pre-overexpression of candidate RNAs in parental cells has been proposed. The miR-122 expression plasmid was transfected into adipose tissue-derived mesenchymal stem cells to obtain miR-122-encapsulated exosomes [106]. The miR-122-loaded exosomes improved the chemosensitivity of hepatocellular carcinoma cells by altering genes such as cyclin G1 and metalloproteinase domain-containing protein 10. In addition, intra-tumoral injection of these miR-122-loaded exosomes showed improved anticancer effects in the xenograft mouse model.

In addition to the pre-overexpression method, as nucleic acids are highly hydrophilic and membrane impermeable, the electroporation method can be used to form pores in the exosomal membranes to facilitate nucleic acids’ entry into exosomes. In particular, 293T cells were transfected with a plasmid expressing the transferrin receptor-binding peptide, T7, to decorate exosomes and antisense miR-21 was loaded by electroporation [107]. The oncogenic miRNA, miR-21, is one of the most highly upregulated miRNAs in solid tumors, including glioblastoma. Therefore, exosomes expressing T7 peptides on their surface passed through the BBB and encapsulating the miR-21 inhibitor were efficiently delivered to transferrin receptor-overexpressed glioblastoma cells. These engineered exosomes showed excellent cancer growth inhibition by suppressing the expression of miR-21.

Similar to natural exosomes, exosome-mimicking technology for nucleic acid delivery has been developed. Nano-sized exosome-mimics were generated by extruding MCF-10A cells through filters of various pore sizes [108]. The yield of exosomes was increased by about 150-folds as aforementioned in the previous section and CDK4 siRNA was loaded by the electroporation method. Exosome-mimics by serial extrusion showed similar efficiencies and safety features to natural exosomes in siRNA delivery, providing another solution for exosome-related barriers in a drug delivery field.

The membrane surface composition of exosomes contributes to their high cellular uptake [122,123,124]. The lipid composition of the exosomal membranes may support cellular internalizations and the proteins on the exosomal surface acting as targeting ligands may play an important leading role for targeted cancer therapies. Based on this concept, biomimetic nanoparticles were developed in which a complex composed of cationic bovine serum albumin and siRNA was coated with an exosomal membrane extracted from breast cancer cells [109]. These biomimetic nanoparticles were superior to the liposome-coated group in in vivo experiments, showing excellent gene silencing effects and inhibition of breast cancer growth.

Besides delivering small RNAs (miRNAs and siRNAs), exosomes could also carry long non-coding RNAs, mRNAs, and circular RNAs. Long non-coding RNAs have attracted much attention due to their high stability and role as miRNA sponges for regulating gene expression. For example, long non-coding circular RNAs, which exhibit a relatively low expression rate in hepatocellular carcinoma patients, were loaded into exosomes derived from HL 7702 cells via a pre-overexpression method [110]. The circular RNAs acted as a sponge for miRNA-331 and inhibited the expression of the BAK1 gene, one of the regulators of apoptosis. Exosomes carrying circular RNAs promoted apoptosis of hepatocellular carcinoma cells and reduced both the weight and size of tumors in xenograft mouse models.

Note that the yield of exosomes is one of the major obstacles in the development of therapeutic exosomes. Most exosome studies are conducted with exosomes derived from primary mesenchymal stem cells or immortalized cells. In contrast, RBCs, which make up about 84% of our body cells, feature high exosome yield. The RBC-derived exosomes have the advantage of avoiding immune responses. Usma et al. have recently reported that anti-miR-125 and Cas9 mRNAs were loaded into exosomes derived from RBCs obtained from a type O donor [43]. Engineered erythrocyte-derived exosomes successfully delivered miRNA and mRNA, demonstrating the efficient miRNA-125 knockdown and gene knockout with CRISPR–Cas9 genome editing in breast cancer mouse models and acute myeloid leukemia models.

### 3.3. Proteins

One of the most promising ways to deliver macromolecular proteins is to utilize exosomes. Proteins can be encapsulated into exosomes via the genetic engineering of donor cells or by direct physical loading such as electroporation.

Donor cells are transfected with a gene encoding the protein of interest. As a result, the cell synthesizes proteins encoded by the inserted genes, which are subsequently secreted into exosomes. The anti-apoptotic protein survivin plays an important role in the viability in multiple cancer cells. The inactive mutant survivin-T34A acts as an inhibitor of this survivin, initiating the mitochondrial apoptotic pathway in cancer cells. It was demonstrated that exosomes derived from melanoma cells overexpressing survivin-T34A by plasmid transfection induce apoptosis and enhance gemcitabine sensitivity in various pancreatic adenocarcinoma cell lines [111]. Exosomes have great potential as vectors for vaccination due to their physiological role played in the immune system and advantage in delivering bioactive molecules. Among the exosomes derived from the immune system, DEX have been demonstrated to have the ability to stimulate immune responses comparable to parental DCs. Recently, attempts have been made to load tumor-specific antigens, peptides, and immune stimulants capable of activating host immune responses against tumor cells into DEX. To use the fetal liver protein α-fetoprotein (AFP) as a hepatocellular carcinoma antigen, a mouse DC cell line was infected with a lentivirus encoding the AFP gene [125]. DEX derived from AFP-expressing DCs induced a robust antigen-specific immune response in a mouse model and resulted in both tumor growth inhibition and prolonged survival.

Proteins could also be loaded directly into exosomes. To increase membrane permeability, tyrosinase-related protein-2 (TRP2) was loaded into serum-derived exosomes through a detergent such as saponin or by the electroporation method [112]. Exosomes containing TRP2 were effectively internalized into macrophages and dendritic cells, and fluorescently-labeled exosomes showed strong signals in lymph nodes, which can be utilized for anticancer treatment through immunotherapy.

Besides loading proteins into the hydrophilic inner space, exosomes could also transport therapeutic membrane proteins in the form of their native structures [126]. CD47, a “don’t eat me” signal, is overexpressed on the surface of most cancer cells. CD47 interacts with the signal-regulatory protein alpha (SIRPα) on the surface of phagocytic cells, weakening the ability of macrophages to engulf cancer cells [127]. Therefore, SIRPα or CD47-binding proteins can be competitive inhibitors to enhance the phagocytosis of phagocytic cells. Based on this mechanism, exosomes expressing SIRPα on their surface to inhibit CD47 functions of tumor cells were developed [113]. SIRPα on the exosome surface enhanced the phagocytic ability of bone-marrow-derived macrophages and effectively inhibited cancer growth in a tumor xenograft model. Another study has shown that exosomes isolated from two mouse tumor cell lines, namely CT26 and TA3HA, transfected with plasmids capable of expressing the exogenous human antigen hMUC1. Results revealed that exosomes expressing the target MUC1 antigen, as well as Hsc70 and MHC class I molecules on their surfaces, induced an effective immune response and inhibited the growth of hMUC1-expressing tumor cells in mice [114]. These studies provide new insights that exosomal membrane scaffolds may be an attractive delivery system for cancer therapy via membrane proteins.

## 4. Exosome Membrane Modifications for Specific Targeting

Considering that exosomes are secreted by cells, they contain cell adhesion molecules, ligands, and lipids that are endogenously expressed in parent cells. Several studies suggest that exosomes have the ability to target their parent cells [103,128]. However, most tumor-targeting studies with in vivo experiments show unexpected results probably due to the heterogeneity of exosomes [129]. These unexpected results are attributed to the change in the composition of solids such as fluids and membrane proteins within exosomes through size heterogeneity caused by uneven invagination. Therefore, to achieve effective tumor targeting, exosomes need to be further optimized with surface engineering. This section focuses on strategies for improving the tumor-targeting of exosome-based drug delivery using two methods: passive and active targeting (Table 3).

### 4.1. Passive Tumor Targeting

Passive tumor targeting is the result of the enhanced permeability and retention (EPR) effect due to increased permeability of the tumor vasculature and ineffective lymphatic drainage. However, EPR effects are a controversial concept, with drug delivery efficiencies of less than 1% even in xenograft tumor models [141]. The heterogeneous size of endothelial gaps, inadequate blood perfusion, and dense interstitial tumor matrix reduce tumor targeting efficiency [142].

Therefore, efficient passive tumor targeting may be an important prerequisite for tumor cell-targeting systems via systemic administration. Strategies to enhance the EPR effect include prolonging the circulation time of exosomes and promoting their extravasation [143]. As in the example presented in the previous section, when CD47, which is a "don’t eat me" signal, was decorated on the exosomal membrane, the circulation time of exosomes was increased by three-folds, resulting in the avoidance of the phagocytosis of macrophages [130,144]. Immune evasion through PEGylation of the exosomal surface also reduced exosome clearance [131]. Copper-64 labeled PEGylated exosomes showed better tumor accumulation and reduced the hepatic clearance of exosomes than native exosomes. Although tumorigenic vessels are leaky and compatible with exosome extravasation, targeting endothelial cell surface molecules may enhance tumor penetration. The α_v_ integrin-specific iRGD (CRGDKGPDC) peptide, which is highly expressed in endothelial cells, was designed to be expressed with the exosomal membrane protein (Lamp2b) on exosomes [132]. These Dox-loaded and iRGD-modified exosomes were injected intravenously and inhibited tumor growth without overt toxicity.

### 4.2. Active Tumor Targeting

Modification of the exosomal membrane affects their biodistribution and specific targeting ability in vivo. Exosomes can be modified to express various extrinsic targeting ligands and stimuli-responsive components, as well as intrinsic targeting ligands such as glycans and integrins.

A fusion protein-based exosome engineering strategy for the treatment of chronic myelogenous leukemia expressing extrinsic targeting ligands has been developed [133]. Although the 5-year survival rate of patients with chronic myelogenous leukemia has been greatly improved with imatinib, a tyrosine kinase inhibitor, drug resistance and long-term side effects still occur due to the non-specific localized accumulation of the drug. Therefore, the interleukin-3 receptor (IL3-R) that overexpressed on the surface of chronic myelogenous leukemia blasts is a good candidate molecule for tumor targeting. HEK cells with high transfection efficiency and high exosome production capacity were transfected with a plasmid encoding the Lamp2b-IL3 fusion protein. Exosomes modified with IL3-targeting ligands were further loaded with imatinib or breakpoint cluster region-Abelson (BCR-ABL) siRNA. Compared with native exosomes, modified exosomes overcame drug resistance and showed enhanced cytotoxicity. Furthermore, engineered exosomes exhibited significantly greater intratumoral accumulation compared to native exosomes in immunodeficient mice bearing subcutaneous chronic myeloid leukemia tumors.

Another engineering strategy to incorporate targeting ligands into exosome membranes is to use click chemistry such as copper-catalyzed azide-alkyne cycloaddition. The specific ligands of interest can be attached to the exosomal surfaces without the use of solutions which can damage the biological features of exosomes by click chemistry that can attach ligands [145,146]. Moreover, click chemistry-based exosome engineering is more efficient than conventional cross-linking methods [147]. The intracellular delivery of proteins could be enhanced by exosome engineering via click chemistry. L-azidohomoalanine-treated B16F10-derived exosomes were conjugated to biotin using a dibenzobicyclooctine (DBCO) polyethylene glycol (PEG)_4_ construct [134]. These exosomes were passively loaded by incubation with horseradish peroxidase conjugated to streptavidin, a natural biotin ligand. The engineered exosomes were able to deliver functional streptavidin-HRP into B16F10 cells with a six-fold higher uptake level compared to free-protein incubation.

Besides adding targeting ligands to the exosome surface, it is also possible to alter the targeting ability of exosomes by removing endogenous surface molecules. Glycosyltransferases, which control the composition of glycome, are strongly associated with cancer progression [148,149]. Glycosylation of lipids and proteins on cellular surfaces plays an important role in cancer metastasis and glycan sialic acid, which is deeply involved in cancer metastasis, was found on the surface of exosomes [150,151]. Another study showed that exosomes derived from liver progenitor cells treated with neuraminidase, an enzyme that removes terminal sialic acid residues, were rapidly accumulated in the lung when intravenously injected into a mouse model compared with the intact EVs that accumulated in the liver [135]. The authors suggested that the modification of glycosylation on the EV surfaces could be used to control their interaction with other cells as well as could induce changes in the distribution of EVs in the body. In addition, after subcutaneous injection of the enzyme-treated exosomes, exosomes were highly accumulated in the axillary lymph nodes. These results suggest that the method of removing the intrinsic surface ligand of exosomes could also be another means for drug delivery.

Environmentally sensitive functional peptides as stimuli-responsive components could also be added to the exosome surface to confer targeting functions. The tumor microenvironment is mildly acidic (pH 5.6 to 6.8) due to glycolysis, hypoxia, and insufficient blood perfusion [152,153]. Therefore, tumor targeting that exploits these features of the tumor microenvironment could be a promising option for designing exosome engineering strategies. 3-(diethylamino) propylamine (DEAP), which binds to the lipid membranes at pH 7.4 but disrupts the membrane below pH 7.0, has been applied to exosomes to allow for the drug release in tumor microenvironments [136]. Indeed, DEAP-engineered exosomes showed enhanced drug release at pH 6.5 compared to the pH 7.4 environment and resulted in increased tumor accumulation. A significant reduction of tumor volume was also observed in HCT-116 human colorectal carcinoma tumor-bearing mice. In addition to exploiting specific intrinsic features of the tumor microenvironment, exosomes can also be engineered for external stimulus-guided targeting, such as magnetism. An exosome in which superparamagnetic nanoparticles are conjugated to reticulocyte-derived exosomes overexpressing transferrin receptors has been developed [154]. The engineered exosomes showed strong responses to external magnetic fields and excellent targeting ability, and inhibited tumor growth in a mouse model by efficiently delivering loaded Dox to cancer cells. This pilot study explored the potential of magnetic field-driven targeting of exosomes, laying a theoretical and experimental basis for future applications in cancer therapy.

## 5. Challenges and Perspectives

Exosomes offer promising aspects to enhance the delivery of therapeutics due to their unique properties such as their endogenous origin and tissue tropism. Moreover, exosomes could be easily engineered to improve drug loading efficiency and targeting capabilities. Despite these advantages of exosomes, detailed understanding related to the exosome biology is still in its infancy and there is much work to be done in the future.

For the therapeutic applications, the choice of exosome source must be made very carefully. Tumor-derived exosomes show remarkable targeting ability against cancer cells and they are loaded with bioactive cargoes that have potential to directly or indirectly promote cancer growth [155,156]. In this regard, techniques to identify and remove or add exosomal components are critical for exosome-based drug delivery for cancer treatment, which might enable us to address barriers arising from heterogeneous exosome subpopulations in the future.

Recently, various strategies such as incubation, transfection, sonication, and electroporation have been developed for loading therapeutic cargoes into exosomes. However, current exosome-cargo-loading strategies are not sufficient to satisfy the loading efficiency required for clinical applications. In particular, the simple incubation method is very limited in the type of cargo to be loaded and the efficiency is too low to be utilized in clinical applications. Transfection methods should further simplify the process and reduce the cost of mass production. The current physical treatment, such as electroporation, is the best method for loading nucleic acids such as siRNA or miRNA into exosomes. However, as this process could induce the aggregation and degradation of charged nucleic acids, as well as could change the properties of exosomes, new approaches are needed [157].

Another major obstacle for the clinical application of exosomes is their low yields. In most preclinical experimental studies, exosomes are obtained through cell culture. Although there are some differences depending on the type of donor cells, less than 1 μg of exosomal protein is produced per ml of culture, which is a level that requires the culturing of huge amounts of cells for clinical trials [29,158]. To overcome this limitation, producing exosomes-mimetic nanovesicles (EMNVs) can be an alternative strategy [94,159,160,161]. EMNVs are produced by extruding cells through sequential micrometer-sized filtration. With these serial extrusion methods, the yield of EMNVs is increased by approximately 100-folds [161,162] and anticancer drugs can be encapsulated simultaneously in this process [94]. However, it is necessary to clarify the changes of in vivo pharmacokinetics/ pharmacodynamics (PK/PD) because the composition of the vesicle membrane can be mixed during the cell extrusion process [33]. In addition, research groups have also developed exosome-liposome hybrid nanoparticles (hybrid EMNVs) in which exosomes and synthetic liposomes are mixed. The representative methods for formulating hybrid EMNVs are, briefly, (1) freeze-thaw [163], (2) simple incubation [164,165], and (3) extrusion [166]. According to study from Lin et al., the authors loaded Cas9 encoding plasmid vectors to liposomes and then incubated it with sgRNA-loaded exosomes isolated from HET293 cells to make hybrid EMNVs. This hybrid form of exosome-like vesicles delivers the CRISPR/Cas9 system to MSCs and induces successful cleavage of target genes [164]. In addition, to overcome the barrier of low yield, some studies using exosomes extracted from various foods are being attempted [85,167,168]. Naturally, these food-derived exosomes are attracting attention because they are generally safe and have excellent cellular uptake efficiency. In particular, milk-derived exosomes showed a 1000-fold higher yield as compared to those derived from animal cell cultures. In addition, oral administration of milk exosomes exhibited improved intestinal absorption [169,170].

In addition to their potential as drug carriers, exosomes have unlimited potential as biomarkers for cancer diagnosis and prognosis. Indeed, numerous studies have been attempted to explore the various profiles and functions of exosomes and to facilitate their clinical applications [171,172,173,174,175]. The potential of exosomes isolated from various body fluids such as blood, saliva, and urine as cancer biomarkers is based on the capture of abnormal cell physiology, but can be expressed differently depending on the source and how to accurately capture unique signals is a major challenge. In addition to this, recent advances in mass spectrometry, next-generation sequencing, and bioinformatics tools have led to a movement to treat cancer with a systems biological approach through detailed proteomic, transcriptomic, glycomic, lipidomic, metabolomic, and genomic analysis of exosomes [176,177]. Identifying correlations between genes involved in exosome biogenesis and tumorigenesis through a systems biology approach and applying them to cancer treatment could be a promising strategy.

Recently, numerous studies have demonstrated that exosomes could be utilized in cancer immunotherapy. Tumor-derived exosomes are considered as a double-edged sword considering they contain both antigens to induce anticancer immunity and factors that can induce cancer progression. Recently, to overcome the risk of such tumor-derived exosomes, exosomes extracted from antigen-presenting DCs have also been utilized. [178,179]. For example, researchers found that peptide-pulsed DC-derived exosomes containing MHC-peptide complexes and co-stimulatory molecules on their membranes could prolong antigen presentation and enhance immunity compared to antigen-presenting DCs [180]. In addition, exosomes are promising candidates for evoking anticancer responses as immunogenic cell death inducers [132]. Recently, DNA-containing exosomes have reported to promote T cell priming and infiltration to trigger a tumor-specific immune response [181,182]. As exosomes are more stable and easier to engineer than activated antigen presenting cells, their clinical potential in cancer immunotherapy is attracting attention.

## 6. Conclusions

With their low immunogenicity and strong biocompatibility, exosomes have heralded a new chapter in drug delivery. Conventional delivery methods of anticancer agents, nucleic acids, and proteins for cancer treatment often fail to achieve desired effects due to in vivo degradation of the therapeutic agent and the lack of targeting ability. Taking advantage of the intrinsic advantageous properties of exosomes, numerous studies have demonstrated that exosomes can be used as carriers for drugs or engineered for anticancer therapy. Although several challenges and obstacles in building a commercial exosome-based drug delivery system remain to be elucidated, an understanding of the detailed biological mechanisms of exosomes and further clinical studies will allow them to emerge as a next-generation nanoplatform for cancer therapy.

## Figures and Tables

**Table 1:** Different types of exosomes originated from various sources and their properties.

Types	Source	Features	Limitations	References
Cell-secreted exosomes	Human embryonic kidney (HEK) cells	Membrane resemblances to various tissues in our body Immunologically inert High efficiency in transfection	Low yield compared to body fluid and food-derived exosomes	[31,32]
	Cancer cells	Tropism toward their parent cells	Less ideal pharmacokinetic profile Lack of studies for metastatic role of cancer cell exosomes	[33,34,35]
	Immune cells (e.g., dendritic cells)	Strictly defined molecular compositions Expression of NK cell-stimulating ligand peptides Surface modification using targeting moieties	Lack of understanding of DEX components (e.g., mRNAs, miRs, and cytokines) and mechanisms regarding how these factors interact with acceptor cells	[36,37,38,39,40]
	Stem cells (e.g., mesenchymal stem cell)	Easy availability from ethically acceptable tissues Large capacity for ex vivo expansion	Similar to other types of exosomes (e.g., lack of PK database and need to improve production efficiency)	[41,42,43]
Blood-derived exosomes	Blood (red blood cells)	Relatively high yield from a single blood unit Reduced unexpected mutations from cell culture No occurring horizontal gene transfer Non-toxic/immunogenic by matching blood types High transfection efficiency	Not determined	[43,44]
Food-derived exosomes	Milk-derived exosomes	Dosing through less-invasive oral cavity Functionalized by simple post-insertion	Variation in shape, size, and cargo contents of exosomes depending on the diet and condition of the source Less understanding of the endogenous biological cargo of milk exosomes	[45,46,47,48]
	Edible plants-derived exosomes (e.g., ginger, grapes, lemon, etc.)		Limited knowledge of cellular molecular properties of PDEs	[49,50,51]

**Table 3:** Exosome modification strategies to improve exosome tumor targeting efficiency.

Targeting Type	Examples	Target	Effect	Exosome Source	Delivery Molecule	Reference
Passive	CD47 surface decoration	SIRPα	Increased exosome circulation time	Mouse embryonic fibroblasts (MEFs)	mRNA	[130]
	Surface PEGylation	N/A	Reduced exosome clearance Enhanced tumor penetration	4T1 murine breast cancer cells	N/A	[131]
	iRGD peptide fusion with Lamp2b	N/A	Inhibited tumor growth without overt toxicity	Mouse immature dendritic cells	Doxorubicin	[132]
Active	IL3-Lamp2b expressing exosome production	IL3 receptors	Increased intratumoral accumulation	HEK293T cells	Imatinib, BCR-ABL siRNA	[133]
	Exosome azide integration, DBCO-PEG4-biotin avidin conjugation	Biotin receptors, glycan biosynthesis process	Higher uptake levels	B16F10 cells	Streptavidin-HRP	[134]
	Neuraminidase	Terminal sialic acid residues from glycoproteins	Rapid accumulation of EVs in the liver	MLP29 cells	N/A	[135]
	3-(diethylamino) propyl-amine (DEAP)	N/A	pH sensitive uptake	RAW 264.7 cells	N/A	[136]
	αCD3 and αHER2 antibodies’ surface expression	CD3, HER2 receptors	Increased T cell activation	Expi293 cells	N/A	[137]
	anti-SSTR2 mAb	Somatostatin receptor 2 (SSTR2)	High toxicity of cancer cells	HEK 293 cells	Romidepsin	[138]
	DSPE-PEG biotin and avidin conjugation	Biotin receptors	Increased stability and encapsulation efficiency	HUVECs	N/A	[139]
	Diacyllipid–aptamer(sgc8) PEG conjugation	PTK7	Increased cellular uptake	ImDC cells	Doxorubicin	[140]
